# Diastolic dysfunction in the diabetic *continuum*: association with insulin resistance, metabolic syndrome and type 2 diabetes

**DOI:** 10.1186/s12933-014-0168-x

**Published:** 2015-01-13

**Authors:** Ricardo Fontes-Carvalho, Ricardo Ladeiras-Lopes, Paulo Bettencourt, Adelino Leite-Moreira, Ana Azevedo

**Affiliations:** EPIUnit - Institute of Public Health, University of Porto, Porto, Portugal; Cardiology Department, Gaia Hospital Center, Vila Nova Gaia, Portugal; Department of Physiology and Cardiothoracic Surgery, Faculty of Medicine, University of Porto, Porto, Portugal; Department of Medicine, Faculty of Medicine, University of Porto, Porto, Portugal; Department of Internal Medicine, Centro Hospitalar São João, Porto, Portugal; Department of Cardiothoracic Surgery, Centro Hospitalar São João, Porto, Portugal; Department of Clinical Epidemiology, Predictive Medicine and Public Health, Faculty of Medicine, University of Porto, Porto, Portugal

**Keywords:** Insulin resistance, Diabetes, Diastole, Diabetic cardiomyopathy

## Abstract

**Background:**

Diabetes increases the risk of heart failure but the underlying mechanisms leading to diabetic cardiomyopathy are poorly understood. Left ventricle diastolic dysfunction (LVDD) is one of the earliest cardiac changes in these patients. We aimed to evaluate the association between LVDD with insulin resistance, metabolic syndrome (MS) and diabetes, across the diabetic continuum.

**Methods:**

Within a population-based study (EPIPorto), a total of 1063 individuals aged ≥45 years (38% male, 61.2 ± 9.6 years) were evaluated. Diastolic function was assessed by echocardiography, using tissue Doppler analysis (E’ velocity and E/E’ ratio) according to the latest consensus guidelines. Insulin resistance was assessed using the Homeostasis Model Assessment of Insulin Resistance (HOMA-IR) score.

**Results:**

The HOMA-IR score correlated to E’ velocity (ρ = −0.20;p < 0.0001) and E/E’ ratio (ρ = 0.20; p < 0.0001). There was a progressive worsening in E’ velocity (p for trend < 0.001) and in E/E’ ratio across HOMA-IR quartiles (p for trend <0.001). Individuals in the highest HOMA-IR quartile were more likely to have LVDD, even after adjustment for age, sex, blood pressure and body mass index (adjusted OR: 1.82; 95% CI: 1.09-3.03). From individuals with no MS, to patients with MS and no diabetes, to patients with diabetes, there was a progressive decrease in E’ velocity (11.2 ± 3.3 vs 9.7 ± 3.1 vs 9.2 ± 2.8 cm/s; p < 0.0001), higher E/E’ (6.9 ± 2.3 vs 7.8 ± 2.7 vs 9.0 ± 3.6; p < 0.0001) and more diastolic dysfunction (adjusted OR: 1.62; 95% CI: 1.12-2.36 and 1.78; 95% CI: 1.09-2.91, respectively).

**Conclusions:**

HOMA-IR score and metabolic syndrome were independently associated with LVDD. Changes in diastolic function are already present before the onset of diabetes, being mainly associated with the state of insulin resistance.

**Electronic supplementary material:**

The online version of this article (doi:10.1186/s12933-014-0168-x) contains supplementary material, which is available to authorized users.

## Background

Subclinical left ventricle diastolic dysfunction (LVDD) is common in the community [1] and is recognized as an important predictor of heart failure [2] and long-term mortality [3]. Current heart failure guidelines [4] give special emphasis to the early detection of these asymptomatic changes of left ventricle function and the identification of its main risk factors.

Epidemiological studies have associated diastolic function with aging, hypertension and myocardial ischemia [1]. Besides, more recent data have also demonstrated an independent association between diastolic function and obesity [5], especially with abdominal obesity [6] and visceral fat mass [7]. Insulin resistance can be one of the important pathophysiological links involved in this association [8,9]. Several studies have suggested that LVDD is one of the earliest signs of myocardial involvement in type 2 diabetes mellitus (T2DM) [10], being a key component of diabetic cardiomyopathy [11].

More recently, it was suggested that changes in diastolic function precede the onset of diabetes, being already present in pre-diabetic patients [12,13], which could be associated with the state of insulin resistance. Metabolic syndrome (MS), or insulin resistance syndrome, is a cluster of cardiovascular risk factors shown to act synergistically to increase the risk of adverse cardiovascular events [14], but also inducing subclinical changes in cardiac structure and function. Indeed, patients with metabolic syndrome also have an increased prevalence of LVDD [15,16], frequently with a subclinical course [17].

In this study, our aim was to evaluate, at the population level, the association between insulin resistance and LVDD in different stages of the diabetic *continuum*.

## Methods

### Study population

Participants were selected within the first follow-up of a cohort, representative at baseline of the adult population of Porto, Portugal — the EPIPorto cohort study. In 1999–2003, the cohort assembly was made by random-digit dialing, using households as the sampling frame, followed by random selection of one person aged 18 years or older in each household. Refusals were not substituted within the same household. The proportion of participation was 70%. At baseline, 2485 participants were recruited. Between October 2006 and July 2008, participants aged 45 years or over were eligible to a systematic evaluation of parameters of cardiac structure and function, which included a cardiovascular clinical history, physical examination, detailed anthropometric evaluation, collection of fasting blood sample and a transthoracic echocardiogram. Among 2048 cohort members in the eligible age range at this time, 134 (6.5%) had died, 198 (9.7%) refused to be re-evaluated and 580 (28.3%) were lost to follow-up (unreachable by telephone or post). From this analysis we excluded 73 patients with previous myocardial infarction, percutaneous or surgical revascularization, prior cardiac surgery or significant (moderate to severe) valvular heart disease. Patients with type 1 diabetes (n = 6) were excluded from the analysis.

Written informed consent was obtained from all the individuals and the local ethics committee (Comissão Ética Centro Hospitalar S. João) approved the study. The investigation conforms to the principles outlined in the Declaration of Helsinki.

### Clinical variables definitions

Hypertension was defined as systolic blood pressure (SBP) ≥140 mm Hg or diastolic blood pressure (DBP) ≥ 90 mm Hg at the time of the visit (mean of 3 readings) or use of antihypertensive medication. T2DM was defined as fasting blood glucose ≥126 mg/dl or the patient’s self-reported history of diabetes or use of diabetes medications. Hypercholesterolemia was defined as total serum cholesterol ≥220 mg/dl or the use of lipid- lowering treatment. Obesity was defined as body mass index (BMI) ≥ 30 kg/m^2^ and central obesity as waist circumference >102 cm in men and >88 cm in women. Metabolic syndrome was defined according to the American Heart Association updated National Cholesterol Education Program Adult Treatment Panel III (AHA/NCEP) criteria [18]. Although there are several definitions for metabolic syndrome, we used this definition because it showed the strongest association with cardiovascular disease in the Portuguese population [19].

MS was diagnosed if any 3 of the following were present: central obesity (WC 102 cm in men and 88 cm in women), raised triglycerides (≥150 mg/dL or fibrates intake), reduced HDL-C (< 40 mg/dL in males and 50 mg/dL in females), raised blood pressure (SBP ≥ 130 mmHg or DBP ≥ 85 mmHg or anti-hypertensive treatment) or raised fasting plasma glucose (FPG ≥ 100 mg/dL or previously diagnosed T2DM). We defined 3 populations of interest in the diabetic *continuum*: individuals with no MS, patients with MS without T2DM and patients with T2DM.

### Analytical data

A fasting venous blood sample was obtained in the morning for measurement of glucose, total cholesterol, LDL, HDL, triglycerides and high-sensitivity C-reactive protein (by immunonephelometry). For insulin measurement the blood was immediately centrifuged and the plasma stored at −20°C, for later measurement. Insulin resistance was assessed using the Homeostasis Model Assessment of Insulin Resistance (HOMA-IR) score in subjects without a history of T2DM before inclusion into the study. The HOMA score was calculated from the formula [20]: HOMA-IR = fasting glucose (mg/dl) × insulin (μU/ml)/405.

### Echocardiography data

All echocardiographic studies were acquired using the same equipment (Hewlett-Packard Sonos 5500). Images were stored on videotape for posterior offline analysis by two experienced cardiologists, blinded to clinical data.

Cardiac chambers dimensions, volumes and left ventricular mass were measured according to current recommendations [21], and indexed to body surface area. Diastolic function was assessed according to the recent consensus guidelines on diastolic function evaluation [22] measuring mitral inflow velocities (E-wave, A wave, E/A ratio) and deceleration time (DT) using pulsed-wave (PW) Doppler in the apical four-chamber view. Velocities were recorded at end-expiration and averaged over three consecutive cardiac cycles. Isovolumetric relaxation time (IVRT) was also assessed accordingly. PW tissue-Doppler velocities were acquired at end-expiration, in the apical four-chamber view, with the sample positioned at the lateral mitral annulus, measuring early diastolic (E’) and late diastolic (A’) velocities and calculating the E/E’ ratio. Patients were categorized into normal diastolic function or LVDD grades I (mild LVDD), II (moderate LVDD) and III (severe LVDD), by two independent cardiologists according to the criteria in the consensus guidelines [22]. In case of discordance, each case was discussed individually, and if doubt persisted no grade was endorsed. LV systolic function was evaluated by determination of LV ejection fraction using the modified Simpson’s rule from biplane 4-chamber.

### Statistical analysis

Statistical analyses were performed using STATA version 12 (STATA Corp, TX, USA). Data are expressed as mean ± standard deviation for quantitative variables with normal distribution, as median and 25th and 75th percentiles (P25-P75) for variables with non-normal distribution or as number (n) and percentage (%) for categorical variables. Pearson coefficient (r) was calculated to assess the correlation of two normally distributed continuous variables and the Spearman correlation (ρ) was used when the variables were non-normally distributed. The trend across categorical variables was tested with the *nptrend* command, which is an extension of the Wilcoxon rank-sum test. Univariate and multivariate logistic regression analysis was performed to predict the presence of LVDD. The variables included in the multivariate model based on previous knowledge were age, gender, systolic blood pressure and BMI. An interaction term was added to the model to assess effect modification by sex, which was not confirmed.

## Results

### Patient characteristics

In this study, 1063 individuals were included, 38% were male, with a mean age of 62.4 ± 10.6 years. The prevalence of MS according to the AHA/NCEP criteria was 41.8% and 11.9% had diabetes. Only 16 patients had T2DM without fulfilling the criteria of MS. Table 1 shows the clinical, anthropometric, analytical and echocardiographic characteristics of the study sample. The total prevalence of LVDD in this study population was 23.7%: 14.5% had mild diastolic dysfunction and 9.2% had moderate or severe diastolic dysfunction. In 25 patients (2.4%) it was not possible to determine LVDD grade due to atrial fibrillation or fusion of the E/A mitral flow pattern. Regarding medication use, 24.6% were doing renin-angiotensin axis blockers, 7.1% were taking calcium channel blockers, 12.1% were on diuretics and 22.8% were taking statins.

**Table 1:** **Characteristics of the study participants**

	**Total**
	**n = 1063**
Age, years	62.2 ± 10.6
Male sex, n (%)	394 (37.1)
Cardiovascular risk factors	
Hypertension, n (%)	361 (34.8)
Diabetes, n (%)	123 (11.8)
Dyslipidemia, n (%)	570 (54.9)
Obesity, n (%)	258 (24.3)
Systolic blood pressure, mmHg	133 ± 20
Diastolic blood pressure, mmHg	78 ± 11
BMI, Kg/m^2^	27.5 ± 4.6
Waist perimeter/height, cm/cm	0.58 ± 0.07
Hip perimeter/height, cm/cm	0.64 ± 0.07
Waist-to-hip ratio	0.92 ± 0.08
**Analytical data**	
Total cholesterol, mg/dL	220 ± 52
HDL, mg/dL	62 ± 44
LDL, mg/dL	134 ± 52
Triglycerides, mg/dL	152 ± 443
Glucose, mg/dL	104 ± 47
C-reactive protein, mg/dL	0.19 (0.09-0.4)
HOMA-IR score	1.09 (0.62-1.86)
**Echocardiography**	
Septum, mm	8.7 ± 1.5
Posterior wall, mm	7.9 ± 1.3
LV mass index, g/m^2^	80.2 ± 21.0
Left atrium volume index, ml/m^2^	29.2 ± 10.4
LV end-diastolic volume, ml/m^2^	66.5 ± 16.9
LV end-systolic volume, ml/m^2^	27.3 ± 10.2
Ejection fraction, %	60.2 ± 6.8
E wave, cm/s	71.4 ± 15.8
A wave, cm/s	78.6 ± 19.9
E/A ratio	0.96 ± 0.32
Deceleration time, ms	238.1 ± 56.9
IVRT, ms	92.0 ± 16.1
E’ velocity, cm/s	10.5 ± 3.3
E/E’ ratio	7.4 ± 2.7
LVDD grade	
Normal, n (%)	792 (76.3)
Mild, n (%)	151 (14.5)
Moderate, n (%)	92 (8.9)
Severe, n (%)	3 (0.3)
Undetermined	25

Data are presented as mean ± standard deviation for continuous variables with normal distribution, median and 25th and 75th percentiles (P25-P75) for variables with non-normal distribution and count (percentage) for categorical variables.

(BMI – body mass index; s – seconds; DD – diastolic dysfunction; IVRT – isovolumetric relaxation time; LV – left ventricle; LVDD – left ventricle diastolic dysfunction).

### Insulin resistance and LVDD

**Figure 1:**
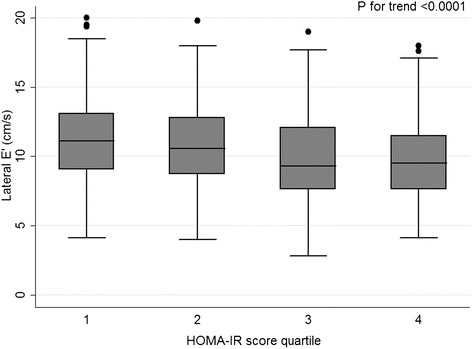
**E’ velocity according to insulin resistance quartiles.**

**Figure 2:**
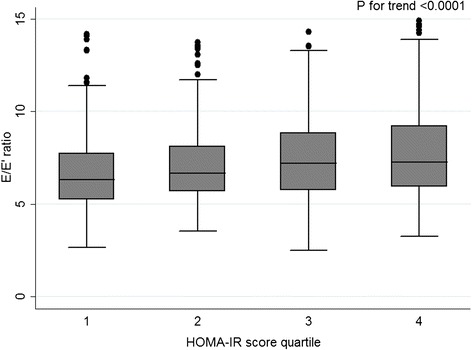
**E/E’ ratio according to insulin resistance quartiles.**

The HOMA-IR score was inversely correlated to lateral E’ velocity (Spearman’s ρ = −0.20; p < 0.001) and positively correlated to E/E’ ratio (Spearman’s ρ = 0.20; p < 0.001). According to HOMA-IR quartiles, higher insulin resistance was associated with lower E’ velocity and higher E/E’ ratio, as shown in Table 2 and Figures 1 and 2.

**Table 2:** **Diastolic dysfunction parameters according to quartiles of insulin resistance and metabolic syndrome status**

	**Diastolic function parameters**			
	**E’ velocity**	**E/E’ ratio**	**E/A ratio**	**DT**
**Insulin resistance**				
**(HOMA-IR score)**				
**Quartile 1**	11.3 ± 3.3	6.8 ± 2.6	1.03 ± 0.37	232.8 ± 52.8
**Quartile 2**	10.7 ± 2.9	7.1 ± 2.3	0.97 ± 0.28	233.2 ± 50.4
**Quartile 3**	10.1 ± 3.6	7.6 ± 2.7	0.92 ± 0.27	240.8 ± 69.5
**Quartile 4**	9.8 ± 3.0	8.1 ± 3.1	0.92 ± 0.35	245.5 ± 54.3
**No Metabolic Syndrome (n = 571)**	11.2 ± 3.3	6.9 ± 2.3	1.01 ± 0.32	232.3 ± 56.9
**Metabolic Syndrome without T2DM (** **n = 331)**	9.7 ± 3.1	7.8 ± 2.7	0.88 ± 0.25	248.4 ± 57.2
**Metabolic Syndrome with T2DM (n = 123)**	9.2 ± 2.8	9.0 ± 3.6	0.95 ± 0.46	237.9 ± 52.7
p for trend	p < 0.001	p < 0.001	p < 0.001	p = 0.002

DT - deceleration time; T2DM - type 2 diabetes mellitus; HOMA-IR - Homeostasis Model Assessment of Insulin Resistance.

Results are presented as mean ± standard deviation.

We observed a stepwise increase in HOMA-IR score according to the grades of diastolic function. HOMA-IR score increased from 0.95 (P25-75: 0.56-1.69) in individuals with normal diastolic function, to 1.30 (P25-75: 0.70-2.03) in patients with grade I diastolic dysfunction and to 1.59 (P25-75: 0.83-2.41) in patients with moderate/severe diastolic dysfunction (p < 0.001).

Table 2 shows the association of diastolic function parameters with insulin resistance (HOMA-IR) and diabetes status. We observed a significant trend for lower E’ velocity and higher E/E’ ratio across HOMA-IR quartiles. Compared to the first quartile, individuals in the highest HOMA-IR quartile showed a 1.82-fold increased odds of LVDD (95% CI: 1.09-3.03), as detailed in Table 3.

**Table 3:** **Crude and adjusted odds ratios for the presence of any grade of diastolic dysfunction according to quartiles of insulin resistance and metabolic syndrome status**

	**Prevalence of LVDD n (%)**	**Crude OR (95% CI)**	Adjusted OR* (95% CI)
**Insulin resistance**			
**(HOMA-IR score)**			
**Quartile 1**	35 (14.9%)	Reference	Reference
**Quartile 2**	42 (18.6%)	1.30 (0.80-2.13)	1.08 (0.63-1.86)
**Quartile 3**	70 (29.3%)	2.37 (1.50-3.73)	1.88 (1.12-3.14)
**Quartile 4**	89 (30.6%)	2.52 (1.63-3.90)	1.82 (1.09-3.03)
**No Metabolic Syndrome (n = 571)**	93 (16.3%)	Reference	Reference
**Metabolic Syndrome without T2DM (n = 331)**	108 (32.6%)	2.54 (1.85-3.50)	1.62 (1.12-2.36)
**Metabolic Syndrome with T2DM (n = 123)**	45 (36.6%)	3.04 (1.98-4.67)	1.78 (1.09-2.91)

T2DM: type 2 diabetes mellitus; LVDD: left ventricular diastolic dysfunction; HOMA-IR - Homeostasis Model Assessment of Insulin Resistance;

OR (95% CI) – odds ratio with 95% confidence interval.

*Variables included in the model: age (continuous), sex, systolic blood pressure (continuous) and body mass index (continuous).

### Metabolic syndrome, T2DM and LVDD

First, we observed a significant increase in HOMA-IR score from patients without MS (0.80; P25-75: 0.44-1.28) to patients with MS without T2DM (1.60; P25-75: 0.91-2.25) and to patients with MS and T2DM (2.56; P25-75: 1.55-4.64), p < 0.001.

Patients with MS or T2DM showed lower E’ velocity and increased E/E’ ratio. Furthermore, as shown in Table 2, there was a significant trend for progressively lower E’ velocity and E/A ratio and higher E/E’ ratio and DT when comparing individuals without MS, to patients with MS not including T2DM and to patients with MS and T2DM.

The prevalence of diastolic dysfunction was 16.3% in the patients without MS, 32.6% in patients with MS and no T2DM and 36.6% in patients with MS and T2DM (p for trend < 0.001; Table 3). After adjusting for age, sex, SBP and BMI, patients with MS and no T2DM showed a 1.62 (95% CI: 1.12-2.36) increased odds of having LVDD and patients with T2DM showed an OR of 1.78 (95% CI: 1.09-2.91). There was no statistically significant difference in the odds of LVDD between patients with MS and no T2DM compared to patients with T2DM (p = 0.696). Also, T2DM was not associated with an increased odds of LVDD (adjusted OR: 1.38; 95% CI: 0.88-2.16) after including in the comparator group both patients with and without MS. We did not find any significant interaction according to gender for the association between diastolic dysfunction with insulin resistance, metabolic syndrome or T2DM.

## Discussion

In this population-based study, we showed that insulin resistance is associated with left ventricular diastolic dysfunction. There was also a progressive worsening of diastolic function parameters (E’ velocity and E/E’ ratio) from individuals without MS, to patient with MS without T2DM and to patients with fully established T2DM. Metabolic syndrome was significantly associated with increased risk of diastolic dysfunction, independently of age, blood pressure and body mass index.

### The association between diastolic dysfunction and insulin resistance

In most patients with glucose metabolism disturbances, insulin resistance is the key pathophysiological mechanism. In this study we found that individuals with higher insulin resistance had worse diastolic function parameters and a significantly increased risk of LVDD, which was independent of other determinants of diastolic function. Until now, few other studies have analyzed the association between insulin resistance and changes in cardiac function, namely with diastolic dysfunction. In a group of selected non-diabetic patients undergoing elective coronary angiography, Dinh et al. also found that insulin resistance was independently associated with LVDD [23] and the same has been observed in a small study of patients with aortic valve sclerosis [24]. Two other studies have demonstrated changes in diastolic function across the diabetic *continuum*, including in pre-diabetic patients [12,13]. Altogether these data suggest that subclinical changes in myocardial diastolic function are already present before the onset of T2DM, being associated mainly with the state of insulin resistance and not only to sustained hyperglycemia.

Metabolic syndrome, also known as insulin resistance syndrome, is common, affecting more than 20% of the adult population of United States and Europe [25]. Insulin resistance is central to the pathophysiology of MS, being associated with a pro-inflammatory, pro-thrombotic and oxidative state and increased risk of atherosclerotic cardiovascular disease [14]. In this study we showed that MS is also independently associated with subclinical changes in myocardial function, namely with LVDD. Compared to individuals without MS, patients with MS had worse diastolic function parameters, including reduced E’ velocity, which is a marker of LV relaxation, and higher E/E’ ratio, which reflects increased LV filling pressures. Moreover, MS was associated with a 1.62-increased odd of LVDD independently of age, sex, blood pressure and body mass index. These data are in accordance with the observations of other smaller studies [15,16,26,27], that also showed a progressive worsening of diastolic function parameters according to the number of criteria for metabolic syndrome [15,16]. Several pathophysiologic mechanisms can be involved in the association between insulin resistance and LVDD [28]. In the heart, insulin stimulates glucose uptake and oxidation and, although it increases fatty acid uptake, it inhibits fatty acid utilization for energy. Therefore insulin resistance results in a reduction of myocardial energy supply due to changes in substrate utilization from glucose to free fatty acids [11,29]. Other involved mechanisms include increased myocardial interstitial fibrosis [30], activation of sympathetic nervous system [31], increased afterload and impaired ventricular-vascular coupling due to arterial stiffness [32,33], endothelial dysfunction [34], increased myocardial oxidative stress [35] or secretion of fatty acid-binding protein 4 [36].

### Diastolic dysfunction and type 2 diabetes mellitus

At the other end of the diabetic *continuum*, it is suggested that diabetes can affect cardiac structure and function in the absence of changes in blood pressure or coronary artery disease, a condition called diabetic cardiomyopathy [11,37]. In humans, LVDD is considered the earliest manifestation of diabetic cardiomyopathy, preceding the development of systolic dysfunction [11]. In our study, we observed that the greatest difference in diastolic function parameters occurs between individuals without MS and patients with metabolic syndrome. However, patients with T2DM had an additional worsening in diastolic function parameters, such as E’ velocity and E/E’ ratio, and a further increased prevalence of LVDD. It is known that the pathogenesis of diabetic cardiomyopathy is multifactorial [11] and beyond the changes associated with insulin resistance, sustained hyperglycemia also increases glycation of interstitial proteins such as collagen by deposition of advanced nonenzymatic glycation end products (AGE) in the extracellular matrix [38], resulting in a further increase in myocardial stiffness. Reinforcing the possibility of an additional “glucotoxic” effect of hyperglycemia on cardiac function, a large study of patients with type 1 diabetes – where insulin resistance is not an important pathophysiological mechanism – showed that incident heart failure was associated with HbA1c and the rate of glycemic control [39].

### Future research and implications to clinical practice

Subclinical LVDD is recognized as an important predictor of heart failure [40] and long-term mortality [1,41]. Therefore, the early identification and correction of the main determinants of subclinical diastolic dysfunction, such as insulin resistance, can be important to reduce morbidity and mortality in these individuals [4]. This can be especially important in the prevention of heart failure with preserved ejection fraction (also known as diastolic heart failure), a disease where no therapy has been shown to significantly improve the prognosis [42].

All these data suggest that the deterioration of diastolic function is already present in an early phase of glucose disturbance metabolism, before the onset of diabetes, being mainly associated with insulin resistance and not only with sustained hyperglycemia. Interestingly, several studies have shown that insulin resistance, with or without diabetes mellitus, predicted incident heart failure independently of other established risk factors [8,43,44]. On the contrary, only few studies [45] have clearly demonstrated an independent association between diabetes and incident heart failure, especially because this association is confounded by the simultaneous presence of other risk factors. Most of these studies have compared diabetic versus non-diabetic individuals, including in the comparator group individuals with insulin resistance and metabolic syndrome, which can partly attenuate the differences in heart failure risk.

Future research will determine if the administration of drugs that increase insulin sensitivity can improve myocardial structure and function, particularly diastolic function. Recently, in animal models of insulin resistance, metformin reduced myocardial fibrosis, attenuated cardiac remodeling and the progression to heart failure [46,47]. This “cardioprotective” effect of metformin can be due to the interference with TGF-beta signaling pathway and activation of the AMP-kinase signaling cascade [48,49]. A new phase II clinical trial is now evaluating if the administration of metformin improves diastolic function in patients with metabolic syndrome and LVDD [50].

Finally, both insulin resistance and metabolic syndrome are closely associated with obesity. Recent data have demonstrated an independent association between LVDD and obesity [5], especially with abdominal obesity [6] and visceral fat mass [7]. Therefore, it has been proposed that insulin resistance was one of the important pathophysiological links involved in this association between obesity and LVDD [8,9]. Our data also show that the association between insulin resistance and LVDD is independent of body mass index, which is in accordance with the study by Ayalon et al. [27].

### Strengths and limitations

Strengths of this study include the relatively large sample of individuals from the general population without other cardiac diseases and the contemporaneous assessment of cardiac diastolic function using tissue Doppler and using the integrated consensus criteria for diastolic dysfunction evaluation [22]. The latest consensus recommendations on LV diastolic function assessment strongly advise in favor of the systematic use of tissue Doppler-derived early mitral annulus velocity (E’ wave) and E/E’ ratios, as the main echocardiographic parameters for diastolic function evaluation. It is known that the E’ wave is a preload-independent index of LV relaxation, being closely related with invasively determined tau (the time constant of isovolumic pressure decline). Moreover, an E/E’ ratio > 15 strongly correlates with invasively determined increased LV filling pressures [22]. On the contrary, E/A ratio and DT, which are derived from the evaluation of mitral inflow velocities, have several limitations in the evaluation of diastolic function, especially because they are dependent on loading conditions and on heart rate. Moreover, these two variables have a U-shaped relation with the severity of diastolic dysfunction, which explains why E/A ratio does not decrease stepwise according to the groups of insulin resistance.

The main limitation is the cross-sectional design, which partially limits comments on causality, as this would be more robust in a prospective design. Although we have excluded patients with clinical signs of coronary artery disease, we did not perform any stress test to exclude myocardial ischemia, which is one determinant of diastolic dysfunction. In this study, patients were not submitted to oral glucose tolerance test. Finally, detailed analysis of left ventricle function using new strain and strain rates techniques was not performed in this study.

## Conclusion

Insulin resistance and metabolic syndrome are associated with diastolic dysfunction independently of age, blood pressure and body mass index. These data suggest that subclinical changes in myocardial diastolic function are already present before the onset of diabetes, being associated mainly with the state of insulin resistance and not only to sustained hyperglycemia. Future research will determine if improving insulin resistance using insulin-sensitizers or lifestyle changes can improve diastolic function.
